# Comprehensive analysis of NGS and ARMS-PCR for detecting EGFR mutations based on 4467 cases of NSCLC patients

**DOI:** 10.1007/s00432-021-03818-w

**Published:** 2021-10-24

**Authors:** Changlong He, Chengcheng Wei, Jun Wen, Shi Chen, Ling Chen, Yue Wu, Yifan Shen, Huili Bai, Yangli Zhang, Xueping Chen, Xiaosong Li

**Affiliations:** 1 Key Laboratory of Molecular Biology for Infectious Diseases (Ministry of Education), Department of Infectious Diseases, Institute for Viral Hepatitis, The Second Affiliated Hospital, Chongqing Medical University, Chongqing, 400010 China; 2 Clinical Molecular Medicine Testing Center, The First Affiliated Hospital of Chongqing Medical University, Chongqing, 400016 China; 3 Oncology Department, The First Affiliated Hospital of Chongqing Medical University, Chongqing, 400016 China; 4 Department of Urology, Union Hospital, Tongji Medical College, Huazhong University of Science and Technology, Wuhan, Hubei China

**Keywords:** Non-small cell lung cancer (NSCLC), Amplification refractory mutation system PCR (ARMS-PCR), Next-generation sequencing (NGS), Epidermal growth factor receptor (EGFR), Mutation

## Abstract

**Background:**

By comparing the detection rate and type of targeted gene mutations in non-small cell lung cancer (NSCLC) between amplification refractory mutation system PCR (ARMS-PCR) and next-generation sequencing (NGS), the characteristics and application advantages of non-small cell lung cancer detection are explained, providing a basis for clinicians to effectively select the corresponding detection methods.

**Methods and materials:**

The cases of targeted genes for lung cancer were selected from the First Affiliated Hospital of Chongqing Medical University from January 2016 to October 2020. A sample of 4467 cases was selected, and they were diagnosed with NSCLC by Pathological biopsy. Sample sources include surgical resection, bronchoscope biopsy, metastatic biopsy, blood, sputum, cytology of pleural effusion. Among them, 3665 cases were detected by ARMS-PCR technique, and 802 cases were detected by NGS technology. The detection rate and type of ARMS-PCR and NGS techniques for EGFR gene mutations (including exon 18, exon 19, exon 20, exon 21 and so on) in different NSCLC samples were compared, respectively.

**Results:**

The total mutation rate of EGFR gene detected by ARMS-PCR was 47.6% while 42.4% detected by NGS which indicated that there was a significant difference between the two methods in detecting total mutation of EGFR gene (*P* < 0.001). In different exons, the EGFR mutation rate detected by two methods is various. The mutation rate of exon 19 by ARMS-PCR detection was evidently higher than that of NGS detection, while the mutation rate of exons 20 and 21 by ARMS-PCR detection were statistically significantly lower than that of NGS detection. Moreover, the multiple mutation rate detected by NGS was 16.3% which was much higher than the 2.7% detected by ARMS-PCR with statistically different.

**Conclusion:**

It showed that NGS could direct the drug use for the resistant patients. However, some rare loci could be detected by NGS but the importance and directed meaning are still unknown and the number of rare mutations is rare too. Further research on new biomarkers and technique is still needed for early diagnosis, directing drug use and assessing the therapy prognosis.

## Background

Lung cancer is the most common malignancy worldwide as well as the leading cause of cancer death in both sexes all around the world with 1.8 million new cases and 1.6 million deaths in 2012 (Ferlay et al. 2015; Bray et al. 2018), and the incidence and mortality of lung cancer have been increasing year by year. In China, lung cancer is the most common cancer among men and second in women, while the disease has the highest death rate among all cancer (Chen et al. 2015). According to the degree of differentiation and morphological characteristics of lung cancer cells, lung cancer can be classified into two categories: non-small cell lung cancer (NSCLC) and small-cell lung cancer (SCLC), with the former accounting for about 85% of diagnosed lung cancers (Hassanein et al. 2012). NSCLC is the highly malignant tumor, and most patients were at an advanced stage when they had been first diagnosed. The surgical treatment, chemotherapy and radiotherapy were the common treatment methods at some time in the past, however, the chemotherapy and radiotherapy had limited efficacy and did not maximize the benefits of patients. At present, the targeted drug therapy has been applied to the treatment of NSCLC, and has shown good therapeutic effects (Zhou et al. 2016). The in-depth research and clinical use of epidermal growth factor receptor (EGFR) and epidermal growth factor receptor-tyrosine kinase inhibitor (EGFR-TKI) have provided a dawn for the treatment of advanced NSCLC. Patients with EGFR gene mutation are more sensitive to EGFR-TKI (Sholl et al. 2010; Dahabreh et al. 2010). Therefore, the detection of EGFR mutation has become a predictive method in targeted treatment of lung cancer (Penzel et al. 2011). In China, the relationship between the mutation rate, distribution of mutation points of EGFR, and clinicopathological characteristics is different in diverse regions (Zhou et al. 2020). So far, there are no clinical data onto the relationship between EGFR mutations and clinical pathological characteristics in NSCLC patients in southwest of China.

At present, the amplification refractory system-PCR (ARMS-PCR) and next-generation sequencing (NGS) technology are clinically commonly detection methods which were used to detect the targeted gene mutation. There was high sensitivity and specificity in ARMS-PCR technology, and the detection rate of gene mutation was far exceeding the traditional PCR, had become one of the most popular and important techniques in the personalized molecular detection of tumors (Coco et al. 2015). On the other hand, NGS, which was based on the principle of edge synthesis edge sequencing, had the advantages of high flux and diverse detection types, and was increasingly used in clinical lung cancer for targeted gene testing. NCCN had recommended that the using of ARMS-PCR or NGS technology for gene mutation detection should prior to targeted treatment of clinical non-small cell lung cancer. In different scenarios, how to choose the right technology of gene testing is a common clinical problem.

In this research, we have analyzed the clinical pathological characteristics and EGFR gene mutations of 4467 patients with NSCLC in Southwest of China, and then analyzed the correlation between the clinical pathological characteristics and EGFR gene mutations which could help to provide data support for targeted drug use. According to the results of targeted gene testing samples, we have compared the advantages and disadvantages of ARMS-PCR and NGS technology which could help clinicians to select an appropriate gene testing method to choose the right targeted drug for the lung cancer patients effectively.

## Materials and methods

### Patients and tumor tissue samples

Totally, 4467 NSCLC patient’s sample data have been collected who were examined and confirmed NSCLC by histopathology, from January 2016 to October 2020 (4 years and 10 months), in The Frist Affiliated Hospital of Chongqing Medical University. The histopathological diagnosis of each specimen was made by two experienced pathologists. Among them, 3665 patients’ EGFR mutation types were tested by ARMS-PCR with an average age of 63.3 ± 10.7 years, 802 NSCLC patients were tested by NGS with a mean age of 63.5 ± 11.1 years. Patients living in southwest of China for a long time were chosen in this research.

### Sample collection, DNA extraction and mutation screening

Tissue samples include surgical resection, bronchoscope biopsy, metastatic biopsy, blood, sputum, cytology of pleural effusion. The cancer samples were obtained from the respective Clinical Departments. The oncologists required EGFR mutation testing based on the individual clinical situations of each patient to guide treatment. Samples were analyzed by the Clinical Molecular Medicine Testing Center, the First Affiliated Hospital of Chongqing Medical University to determine their EGFR mutation status. The database about the patient’s age, gender, sample types and mutation types was established. NSCLC were classified according to the 2015 WHO classification (Travis et al. 2015).

### ARMS-PCR

DNA was aspirated from formalin-fixed paraffin-embedded (FFPE) afterwards using TRIzol^®^ reagent (cat. no. 15596–026; Invitrogen; Thermo Fisher Scientific, Inc.), according to the manufacturer's protocols. DNA concentrations of all samples were determined using a NanoDrop ND-1000 spectrophotometer at 280 nm (Thermo Fisher Scientific, Inc.). The gene mutations of such samples were detected via amplification refractory mutation system-polymerase chain reaction (ARMS-PCR) and the thermo-cycling conditions of PCR were as follows: 1 cycle of 95 °C for 5 min; followed by 15 cycles of 95 °C for 25 s, 64 °C for 20 s, 72 °C for 20 s; and then finally 31 cycles of 93 °C for 25 s, 60 °C for 35 s, 72 °C for 20 s. The ARMS-PCR reagents were provided by AmoyDx Diagnostics Co., Ltd. (cat. no. 20143402001).

### NGS (Capture-based targeted DNA sequencing)

The genomic DNA profiles of tumor tissue samples were performed using capture-based targeted sequencing. The DNA samples were analyzed with the Qubit dsDNA assay (Thermo Fisher Scientific, Waltham, MA). The library was constructed using 68 gene panel (Burning Rock Biotech Ltd., RS0323F-V2, Guangzhou, China). DNA was fragmented by Covaris M220, then, repaired the end, phosphorylation and adaptor ligation were performed. DNA fragments of 200–400 bp in size were selected by Agencourt AMPure beads (Beckman Coulter, Brea, CA, USA), following by hybridization with capture probes baits. Then, hybridization selection with magnetic beads and polymerase chain reaction (PCR) amplification were performed. The quality and size of the fragments were analyzed. 50 ng of DNA was used for library construction. Twelve PCR cycles were used for library amplification. Then, the indexed samples were sequenced on a miniseq sequencer (Illumina, San Diego, CA) with paired-end reads, and the Miniseq High Output Reagent Cartridge ragent was used. The entire exon regions of EGFR were detected, including single-nucleotide variation (SNV) within ± 20 bp, short insertion or deletion variation (INDEL), gene copy number variation (CNV), and breakpoints occur within the capture range gene rearrangement.

### Statistical analyses

Patients were classified as mutated or wild type based on the presence of EGFR mutations. The quality and quantity of the material were measured by the total number or percentage of neoplastic cells to determine whether the sample could be analyzed. Their association with the clinical data was tested using Fisher’s exact test or a two-sided Chi-square test. A *P* value of 0.05 or less was considered significant. The average age was shown as means ± standard deviation (SD). The NGS data were analyzed using Burning Rock Biotech. The human genome (hg19) was used for FASTQ data mapping using a BWA aligner 0.7.1037. The local alignment optimization, variant calling and annotation were performed, respectively, by brFire system (Burning Rock Biotech Ltd., RS0323F-V2, Guangzhou, China), using GATK3.2, MuTect and VarScan, respectively. In addition, DNA translocation analysis was performed using Factera 1.4.3. All of the statistical analyses were performed with SPSS 24.0 software and the time of EGFR analysis was between November 2020 and March 2021.

## Results

### Detection of EGFR gene mutation in 3665 patients with NSCLC by ARMS-PCR

Among 3665 NSCLC patients (2042 male and 1623 female) using ARMS-PCR, 1744 cases had EGFR gene mutation, and the total mutation rate was 47.6%. Among 2042 NSCLC male patients, 679 cases had EGFR gene mutations, and the mutation rate was 33.3%. 1065 out of 1623 NSCLC female patients were identified to have EGFR activating mutations, with the mutation rate of 65.6%. Among 3665 NSCLC patients, there was statistically significant (*P* = 0) difference between the female and male cases in the mutation rates of EGFR. And patients, whose age ranged from 45 to 59 years old, had the highest EGFR gene mutation rate among patients of all ages, and the gene mutation rate was 50.9% (611/1200), with statistically significant difference among other different age groups (*P* = 0.005) (There was no statistical significance because patients over 90 years old had fewer cases and a large margin of error.) Sample types of all cases included biopsies of diseased tissues, metastatic tissues, cytological examinations, sputum fluids, whole blood and the mutation rates of EGFR gene were, respectively, 48.7% (1032/2118), 48.5% (126/260), 48.4% (522/1078), 28.8% (40/139) and 34.3% (24/70), also with statistically significant difference between different sample types (*P* = 0). The characteristics of patients and EGFR mutation rate are shown in Table 1.

**Table 1:** NCSLC patients EGFR mutation rate and clinical information analysis by ARMS-PCR

Overall patients	Case(*n*)	EGFR		Positive rate (%)	*χ*^2^	*P*
		Mutated type	Wild type			
Sex						
Male	2042	679	1363	33.3	379.833	0.00
Female	1623	1065	558	65.6		
Age						
< 45	200	91	109	45.5	14.302	0.005
45 ~ 59	1200	611	589	50.9		
60 ~ 74	1813	852	961	47.0		
75 ~ 89	447	186	261	41.6		
> 90	5	4	1	80.0		
Sample type						
Tumor tissue	2118	1032	1086	48.7	26.166	0.00
Metastatic tissue	260	126	134	48.5		
Cytology	1078	522	556	48.4		
Sputum	139	40	99	28.8		
Blood	70	24	46	34.3		

### Detection of EGFR gene mutation in 296 patients with advanced NSCLC by NGS

Totally, 802 NSCLC patients (468 male and 334 female) were further detected by NGS. Among them, 340 cases had EGFR gene mutation, and the total mutation rate was 42.2% (340/802). There were 141 male and 199 female in the EGFR gene mutation cases, and the gene mutation rate was 30.1% (141/468) and 59.6% (199/334), respectively, which had statistically significant (*P* = 0) difference. And patients, whose age ranged from 45 to 59 years old, had the highest EGFR gene mutation rate among patients of different ages, and the gene mutation rate was 48% (123/256), without statistically significant difference among different age groups (*P* = 0.13). Sample types of all cases contained biopsies of diseased tissues, metastatic tissues, cytological examinations, sputum fluids, whole blood and the mutation rates of EGFR gene were, respectively, 46.6%(206/442), 33.3%(1/3), 63.3%(31/49), 50%(4/8), 32.7%(98/300). And the difference was statistically significant among different sample types (*P* = 0). All characteristics of patients and EGFR mutation rate are shown in Table 2.

**Table 2:** NCSLC patients EGFR mutation rate and clinical information analysis by NGS

Overall patients	Case(*n*)	EGFR		Positive rate (%)	*χ*^2^ (Bray et al. 2018)	*P*
		Mutated type	Wild type			
Sex						
Male	468	141	327	30.1	69.23	0.00
Female	334	199	135	59.6		
Age						
< 45	32	12	20	37.5	12.087	0.13
45 ~ 59	256	123	133	48.0		
60 ~ 74	379	163	216	43.0		
75 ~ 89	131	42	89	32.1		
> 90	4	0	4	0.0		
Sample type						
Tumor tissue	442	206	236	46.6	24.109	0.00
Metastatic tissue	3	1	2	33.3		
Cytology	49	31	18	63.3		
Sputum	8	4	4	50.0		
Blood	300	98	202	32.7		

### EGFR gene mutation unit types detected by ARMS-PCR in 3665 advanced NSCLC patients

3665 NSCLC patients were detected EGFR Gene Mutation unit types using ARMS-PCR, 1744 cases had mutation units, which including single-site mutation in 1654 patients and multiple locus mutation in 90 patients. Total mutation rate was 47.6%. The total number of positive EGFR mutation units was 1838, due to multiple mutation. The total mutation rates of exon 18, 19, 20 and 21 were 1.7, 20.7, 3.1 and 24.7%, respectively, and their composition ratios were 3.4, 41.2, 6.2 and 49.3%. Among seven mutation types, unit points mutation rate was G719X (1.7%), 19 del (20.7%), S768I (0.7%), T790M (1.1%), 20ins (1.3%), L858R (23.4%), L861Q (1.3%) which has been presented in Table 3. Among these NSCLC patients using ARMS-PCR, exon 19 deletion and exon 21 mutations were the main EGFR mutation types which accounted for 90.5% of total mutation cases. It is of great significance for guiding targeting therapy with TKI in clinic to those patients with advanced NSCLC.

**Table 3:** Characteristics of mutation units types of EGFR in 3665 NSCLC patients analysis by ARMS-PCR

Exons	Sites	Mutations	Amino acid sequence changes	Unit positive case (*n*)	Unit point mutation rate (%)	Total mutation rate of exon (%)	Composition ratio (%)
**18**	3	G719X G719A G719S G719C	2156G > C 2155G > A 2155G > T	62	1.7	1.7	3.4
**19**	19	19del-		757	20.7	20.7	41.2
		E746-A750del	2236-2250del				
		S752-I759del	2254-2277del				
		The rest are not listed					
**20**	5	T790M	2369C > T	39	1.1	3.1	6.2
		S768I	2303G > T	26	0.7		
		20Ins		49	1.3		
		H773-V774insH D770-N771insG V769-D770insASV	2319-2320insCAC 2310-2311insGGT 2307-2308insgccagcgtg				
**21**	2	L858R	2573 T > G	858	23.4	24.7	49.3
		L861Q	2582 T > A	47	1.3		

The mutation of EGFR gene in patients with advanced NSCLC was detected by ARMS-PCR. Unit point mutation rate = the number of mutations per unit point/total number of mutations; total mutation rate of exons = the number of exon mutations/total number of mutations; composition ratio = the number of mutations/total number of mutations

*NSCLC* non-small cell lung cancer, *EGFR*: epidermal growth factor receptor, *§* include 1654 single-site mutation patients and 90 multiple locus mutation patients

### EGFR gene mutation unit types detected by NGS in 802 advanced NSCLC patients

A total 802 advanced NSCLC patients were further detected for EGFR Gene Mutation units types by NGS. Among them, there were 209 single-site mutation patients and 131 multiple locus mutation patients. The total mutation rate was 42.4%, and the single-site and multiple locus mutation rates were 26.1 and 16.3%, respectively. The total mutation rates of exon 18, 19, 20, 21 and gene amplification were 1.9, 15.5, 6.9, 20.9 and 7.2%, respectively, and their composition ratios were 3.5, 29.2, 13.0, 49.3 and 13.7%. Among them, unit point mutation rate was G719X (1.4%), E709 (0.5%), 19del (15.5%), S768I (0.5%), T790M (3.9%), 20Ins (2.0%), L858R (19.5%), L861Q (1.4%) which has been presented in Table 4. Otherwise, there were 5 rare mutations detected including p.R547*, p.R677C, p.E1079K, p.C624Y and EGFR-PPP1R17. In patients with advanced NSCLC detected by NGS, exon 19 deletion and exon 21 mutation were still the main types of EGFR gene mutations, while mutation rates of exon 19 and 21 detected by NGS were significantly lower than which detected by ARMS-PCR. However, the detection of multiple locus mutation was much higher than that of ARMS-PCR which had important clinical significance for better selection of targeted diagnosis and therapy in advanced NSCLC.

**Table 4:** Characteristics of mutation units types of EGFR in 802 NSCLC patients analysis by NGS

Exons	Sites	Mutations	Amino acid sequence changes	Unit positive case (*n*)	Unit point mutation rate (%)	Total mutation rate of exon (%)	Composition ratio (%)
		G719X		11	1.4	1.9	3.5
		G719A	2156G>C				
18	3	G719S	2155G>A				
		G719C	2155G>T				
		E709		4	0.5		
19	19	19del-		124	15.5	15.5	29.2
		E746-A750del	2236-2250del				
		S752-I759del	2254-2277del				
		The rest are not listed					
20	5	T790M	2369C>T	31	3.9	6.9	13.0
		S768I	2303G>T	4	0.5		
		20Ins		16	2.0		
		The rest are not listed		4	0.5		
21	2	L858R	2573T>G	156	19.5	20.9	49.1
		L861Q	2582T>A	11	1.4		
Rare mutation	5			5	0.6		1.2
5		p.R547*					
17		p.R677C					
27		p.E1079K					
15		p.C624Y					
Exon28_Intron4		EGFR-PPP1R17					
Gene amplification				58	7.2		13.7

The mutation of EGFR gene in patients with advanced NSCLC was detected by ARMS-PCR. Unit point mutation rate = the number of mutations per unit point/total number of mutations

*NSCLC* non-small cell lung cancer, *EGFR* epidermal growth factor receptor, *§* include 209 single-site mutation patients and 131 multiple locus mutation patients

### Comparison and analysis of the two methods for detecting mutation rates of EGFR gene

By detecting NSCLC patients with two different ways, ARMS-PCR and NGS, we had compared and analysed the mutation rates of the two methods. Surprisingly, the result had showed that the difference between the EGFR mutation rate in patients determined by ARMS-PCR and those by NGS was significant. Although ARMS-PCR and NGS showed exon 19 deletion and exon 21 mutation were both the main mutation types, the difference of mutation rates was statistically significant. Then we compared the mutation rates of exon 18, 19, 20, 21 and multiple locus mutations by row X list chi-square test. The results showed that the total mutation rates detected by ARMS-PCR were higher than those by NGS (47.6 vs. 42.4%, *P* < 0.05), however, the multiple locus mutation rate detected by ARMS-PCR was much lower than those by NGS (2.7 vs. 16.3%, *P* < 0.001) (Table 5). Except for the difference in exon 18 mutation rate which was similar between the two methods, the mutation rate in exon 19, 20 and 21 had statistical difference. This results suggested that ARMS-PCR had higher detection rate and higher sensitivity than NGS in single-site gene mutation of EGFR, which could meet the needs of clinicians to select the EGFR-TKI targeted drugs quickly and accurately. In contrast, NGS had significant advantages in detecting multiple and rare mutation, which could meet the needs of personalized medicine better.

**Table 5:** Comparison of the two methods for detecting mutation rates of EGFR gene

Relevant factors	Method	Wild type	Mutant type	Mutation rate (%)	*χ*^2^ (Bray et al. 2018)	*P*
Exon 18	ARMS-PCR NGS	3603 787	62 15	1.8 1.9	0.124	0.725
Exon 19	ARMS-PCR	2908	757	20.7	11.21	0.001
	NGS	678	124	15.5		
Exon 20	ARMS-PCR	3551	114	3.1	25.384	0.000
	NGS	747	55	6.9		
Exon 21	ARMS-PCR	2760	905	24.7	5.403	0.020
	NGS	635	167	39.4		
Multiple locus mutations	ARMS-PCR		90	2.7	269.319	0.000
	NGS		131	16.3		
Total	ARMS-PCR	1921	1744	47.6	7.125	0.008
	NGS	462	340	42.4		

Comparison of mutation rates of exon-related mutation sites detected by the two methods. The mutation rates were compared by row X list Chi-square test, and *P*

## Discussion

At present, lung cancer remains one of the most common cancers in China. The mortality and morbidity of lung cancer is still one of the fastest-growing cancers in recent years in China (Dajac et al. 2016). NSCLC accounts for approximately 85% of lung cancer cases, which seriously threatens human health and life (Won et al. 2015). EGFR-TKI is a representative drug, the efficacy of which has been clinically confirmed. However, the sensitivity and prognosis of EGFR-TKI treatment is associated with the status of EGFR gene mutation, and the treatment has a significant impact on sensitive mutant patients, while the prognosis of non-mutant patients is poor (Roskoski 2014; Nakata et al. 2015). So, to promote the therapeutic effect, the EGFR gene mutation status is supposed to be cleared before carrying out the clinical therapy of NSCLC patients (Gazzeri 2018). There are differences in EGFR gene mutations in NSCLC in different regions and different races (Graham et al. 2018).

In this study, the clinical data of 4467 patients with NSCLC had been collected in southwest of China, and the total mutation rate of EGFR gene was 46.7%, similar to the 48.7% EGFR mutation rate reported by the West China Hospital of Sichuan University in China. In our research, the most frequent mutation types of EGFR were exon 19 deletion and exon 21 mutation (L858R), which resembled those that reported in researches performed in the countries of East Asia (Shi et al. 2015; Shi et al. 2014; Liam et al. 2013; Lee et al. 2015). According to previous studies, the EGFR gene mutations in NSCLC patients are related to race, sex, smoking history, pathological type, and sample type (Zhou et al. 2016; Shi et al. 2014; Ma et al. 2020). And this research further confirms that EGFR gene mutations are related to sex and sample type. Therefore, both previous studies and the current study have shown that EGFR mutations are more common to female, never-smoker and lung adenocarcinoma, and this group of people is more likely to benefit from EGFR-TKI drugs, which could help to choose the best therapeutic drugs for clinicians with a significant reference.

At present, ARMS-PCR and NGS are the main methods in EGFR gene mutation detection. The application of NGS is increasing as a kind of new technology which is being used to test inherited disorders and different tumor gene mutations (Yohe and Thyagarajan 2017). NGS has great significance for the further step of personalized medicine by detecting the somatic driver mutations, mutational burden quantification, resistance mechanisms, germline mutations, which also has the ability to sequence circulating tumor DNA (ctDNA) in liquid biopsy for screening and early diagnosis (Morganti et al. 2019; Chen and Zhao 2019). In many cases of lung cancer, NGS could avoid the difficulties by detecting ctDNA, as the biopsy is a painful procedure for patients (Thomas et al. 2013). Many reports have indicated that the advantages of NGS was high sensitivity in detecting actionable alterations used by gene panel (Masago et al. 2015; Rangachari et al. 2015; Shao et al. 2016). Especially in Asian predominant population, NGS represents a non-invasive, cost-effective way of diagnosis compared with sequential testing strategies (Tan et al. 2020). However, challenge follows with promise. For example, the increasing generation of enormous sequence presents a huge challenge for data integration, analysis and interpretation (Xuan et al. 2013). NGS could easily discover numerous genetic variations between tumor and normal tissue, but it will be hard to extract clinically useful and actionable information and validate the significant genotype–phenotype associations (Gerlinger et al. 2012). The related bioinformatics challenge, such as CNVs, SNVs and epigenetic variations, all pose difficulties in detection and are therefore associated with higher error rates (Crowley et al. 2013; Pantel and Alix-Panabières 2013). In clinical practice, NGS also meets the problem of high cost in time and economy. From nuclein extraction to report diagnosis, it will take up 3–5 days and the high expense of machine and reagent make it hard to popularize in the primary hospitals in China.

ARMS-PCR has been widely used since the end of 2015 to replace the traditional Sanger sequencing in domestic hospital to detect the mutation type of EGFR gene which is based on quantitative polymerase chain reaction (qPCR) (Kimura et al. 2006). ARMS-PCR has been approved by the China Food and Drug Administration (CFDA) which is sensitive and reliable for detecting EGFR gene mutation (Liang et al. 2018). The advantages of this method are mature, simple, reliable, and the experimental operation is easy, the detection period is short, which could avoid the toxic operation and detect early genetic alteration that could better meet the current clinical needs (Liu et al. 2015). The reason for ARMS-PCR with the high sensitivity is its peculiar primer design, one pair of primers could amplify a conserved region, and another primer pair could target the point mutation. ARMS-PCR applies only in the detection of known mutations, each reaction system could only detect the pre-specified gene mutation. Therefore, a large amount of DNA samples and primer pairs are needed, if an unknown gene mutation region must be detected and analyzed, which will make the detection method expensive such as NGS. Although the ARMS-PCR is the main mean to detect EGFR gene mutations (Liang et al. 2018), it is considered as an alternative method because the NGS has the advantages of high flux and diverse detection types in detecting gene mutations (Coco et al. 2015).

Learning from our data, the proportion of EGFR mutations detected by such two methods was basically consistent with previous reports, and the main mutations were exons 19 and 21. But there was some statistically significant difference in the composition ratio of exon 19 mutation between NGS and ARMS-PCR with 29.2 vs 41.2% (*P* < 0.05). About mutation rate, the total mutation rate of EGFR gene detected by ARMS-PCR was 47.6% which was higher than that detected by NGS (42.4%). The single mutation rates detected by ARMS-PCR in exons 18, 19, 20 and 21 were 1.8, 20.7, 3.1 and 24.7%, respectively, and the single mutation rates detected by NGS in exons 18, 19, 20 and 21 were 1.9, 15.5, 6.9 and 39.4%. The mutation rate detected by NGS was much higher than that by ARMS-PCR in exons 20 and 21, however, the mutation rate detected by NGS was lower than that by ARMS-PCR in exon 19. The difference was statistically significant, but there was no significant difference in exon 18. Such difference may be associated with the type of sample, the number of subjects, different instruments and reagents. The T790M and 20-ins were resistant mutation units, which suggested that patients with sensitive mutations developed resistance during medical treatment, in this study, there were 70 cases of T790M mutation in 167 patients with resistance after EGFR-TKI treatment, accounting for 41.4%. Surprisingly, the multiple mutation rate detected by NGS was 16.3% which was much higher than the 2.7% detected by ARMS-PCR with statistically different. At present, the standard care for patients that resist TKI drug caused by the EGFR T790M mutation is to use the third-generation TKI drugs which may be important to direct the drug use for the resistant patients using NGS. However, some rare loci can be detected by NGS but the importance and directed meaning are still unknown, and the number of rare mutations is rare too. Therefore, NGS has already been used to identify new biomarker candidates for the early diagnosis of lung cancer and is increasingly used to guide personalized treatment decisions (Kruglyak et al. 2016) and could be used to explore the mechanism of drug resistance.

## Conclusion

Further research is still needed to direct drug use and assess therapy prognosis. With the increasing of drug resistance to the therapy, the EGFR gene mutation should be monitored in patients with poor therapeutic effects when conditions permit. In targeted treatment of lung cancer, NGS has a higher throughput and a wide range of detections than ARMS-PCR technology, and when there are fewer clinical biopsy samples, more information on genetic mutations can be obtained at once, providing a more comprehensive therapeutic basis for the clinic.

## Data Availability

All data generated or analyzed during this study are included in this published article.

## Abbreviations

NSCLC: Non-small cell lung cancer
ARMS-PCR: Amplification refractory mutation system PCR
qPCR: Quantitative polymerase chain reaction
NGS: Next-generation sequencing
EGFR: Epidermal growth factor receptor
ctDNA: Circulating tumor DNA
CFDA: China food and drug administration

## References

[CR1] Bray F et al (2018) Global cancer statistics 2018: GLOBOCAN estimates of incidence and mortality worldwide for 36 cancers in 185 countries. CA Cancer J Clin. 68: 394–424. 10.3322/caac.21492 10.3322/caac.21492 30207593

[CR2] Chen M, Zhao H (2019). Next-generation sequencing in liquid biopsy: cancer screening and early detection. Hum Genomics.

[CR3] Chen W, et al (2016) Cancer statistics in China, 2015. CA Cancer J Clin. 66: 115–132. 10.3322/caac.21338 10.3322/caac.21338 26808342

[CR4] Coco S (2015). Next generation sequencing in non-small cell lung cancer: new avenues toward the personalized medicine. Curr Drug Targets.

[CR5] Crowley E, Di Nicolantonio F, Loupakis F, Bardelli A (2013). Liquid biopsy: monitoring cancer-genetics in the blood. Nat Rev Clin Oncol.

[CR6] Dahabreh IJ (2010). Somatic EGFR mutation and gene copy gain as predictive biomarkers for response to tyrosine kinase inhibitors in non-small cell lung cancer. Clin Cancer Res.

[CR7] Dajac J, Kamdar J, Moats A, Nguyen B (2016). To screen or not to screen: low dose computed tomography in comparison to chest radiography or usual care in reducing morbidity and mortality from lung cancer. Cureus.

[CR8] Ferlay J (2015). Cancer incidence and mortality worldwide: sources, methods and major patterns in GLOBOCAN 2012. Int J Cancer.

[CR9] Gazzeri S (2018). Nuclear EGFR: a new mode of oncogenic signalling in cancer. Biol Aujourdhui.

[CR10] Gerlinger M (2012). Intratumor heterogeneity and branched evolution revealed by multiregion sequencing. N Engl J Med.

[CR11] Graham RP (2018). Worldwide frequency of commonly detected EGFR mutations. Arch Pathol Lab Med.

[CR12] Hassanein M (2012). The state of molecular biomarkers for the early detection of lung cancer. Cancer Prev Res.

[CR13] Kimura H (2006). Detection of epidermal growth factor receptor mutations in serum as a predictor of the response to gefitinib in patients with non-small-cell lung cancer. Clin Cancer Res.

[CR14] Kruglyak KM, Lin E, Ong FS (2016). Next-generation sequencing and applications to the diagnosis and treatment of lung cancer. Adv Exp Med Biol.

[CR15] Lee SH (2015). Analysis of mutations in epidermal growth factor receptor gene in Korean patients with non-small cell lung cancer: summary of a nationwide survey. J Pathol Transl Med.

[CR16] Liam CK, Wahid MI, Rajadurai P, Cheah YK, Ng TS (2013). Epidermal growth factor receptor mutations in lung adenocarcinoma in Malaysian patients. J Thorac Oncol.

[CR17] Liang C (2018). Detection of rare mutations in EGFR-ARMS-PCR-negative lung adenocarcinoma by Sanger sequencing. Yonsei Med J.

[CR18] Liu J, Zhao R, Zhang J, Zhang J (2015). ARMS for EGFR mutation analysis of cytologic and corresponding lung adenocarcinoma histologic specimens. J Cancer Res Clin Oncol.

[CR19] Ma Y (2020). Oncogenic genetic alterations in non-small-cell lung cancer (NSCLC) in Southwestern China. Cancer Manag Res.

[CR20] Masago K (2015). Next-generation sequencing of tyrosine kinase inhibitor-resistant non-small-cell lung cancers in patients harboring epidermal growth factor-activating mutations. BMC Cancer.

[CR21] Morganti S (2019). Next Generation Sequencing (NGS): a revolutionary technology in pharmacogenomics and personalized medicine in cancer. Adv Exp Med Biol.

[CR22] Nakata A (2015). Elevated β-catenin pathway as a novel target for patients with resistance to EGF receptor targeting drugs. Sci Rep.

[CR23] Pantel K, Alix-Panabières C (2013). Real-time liquid biopsy in cancer patients: fact or fiction?. Can Res.

[CR24] Penzel R (2011). EGFR mutation detection in NSCLC–assessment of diagnostic application and recommendations of the German panel for mutation testing in NSCLC. Virchows Arch.

[CR25] Rangachari D (2015). Experience with targeted next generation sequencing for the care of lung cancer: insights into promises and limitations of genomic oncology in day-to-day practice. Cancer Treat Commun.

[CR26] Roskoski R (2014). ErbB/HER protein-tyrosine kinases: Structures and small molecule inhibitors. Pharmacol Res.

[CR27] Shao D (2016). A targeted next-generation sequencing method for identifying clinically relevant mutation profiles in lung adenocarcinoma. Sci Rep.

[CR28] Shi Y (2014). A prospective, molecular epidemiology study of EGFR mutations in Asian patients with advanced non-small-cell lung cancer of adenocarcinoma histology (PIONEER). J Thorac Oncol.

[CR29] Shi Y (2015). Molecular epidemiology of EGFR mutations in Asian patients with advanced non-small-cell lung cancer of adenocarcinoma Histology-Mainland China subset analysis of the PIONEER study. PLoS One.

[CR30] Sholl LM (2010). EGFR mutation is a better predictor of response to tyrosine kinase inhibitors in non-small cell lung carcinoma than FISH, CISH, and immunohistochemistry. Am J Clin Pathol.

[CR31] Tan AC (2020). Utility of incorporating next-generation sequencing (NGS) in an Asian non-small cell lung cancer (NSCLC) population: Incremental yield of actionable alterations and cost-effectiveness analysis. Lung Cancer.

[CR32] Thomas A, Rajan A, Lopez-Chavez A, Wang Y, Giaccone G (2013). From targets to targeted therapies and molecular profiling in non-small cell lung carcinoma. Ann Oncol.

[CR33] Travis WD (2015). The 2015 World Health Organization classification of lung tumors: impact of genetic, clinical and radiologic advances since the 2004 classification. J Thorac Oncol.

[CR34] Won JK (2015). Concomitant ALK translocation and EGFR mutation in lung cancer: a comparison of direct sequencing and sensitive assays and the impact on responsiveness to tyrosine kinase inhibitor. Ann Oncol.

[CR35] Xuan J, Yu Y, Qing T, Guo L, Shi L (2013). Next-generation sequencing in the clinic: promises and challenges. Cancer Lett.

[CR36] Yohe S, Thyagarajan B (2017). Review of clinical next-generation sequencing. Arch Pathol Lab Med.

[CR37] Zhou J (2016). Prevalence and clinical profile of EGFR mutation in non-small-cell lung carcinoma patients in Southwest China. Asian Pac J Cancer Prev.

[CR38] Zhou YC (2020). Analysis of EGFR mutation and clinical features of lung cancer in Yunnan. Zhonghua zhong liu za zhi [Chinese journal of oncology].

